# Toxicity of Bioactive Molecule Andrographolide against *Spodoptera litura* Fab and Its Binding Potential with Detoxifying Enzyme Cytochrome P450

**DOI:** 10.3390/molecules26195982

**Published:** 2021-10-02

**Authors:** Edward-Sam Edwin, Prabhakaran Vasantha-Srinivasan, Sengottayan Senthil-Nathan, Muthiah Chellappandian, Sengodan Karthi, Radhakrishnan Narayanaswamy, Vethamonickam Stanley-Raja, Haridoss Sivanesh, Ramakrishnan Ramasubramanian, Asma A. Al-Huqail, Faheema Khan, Patcharin Krutmuang, Ahmed Abdel-Megeed, Aml Ghaith, Chae-Hoon Paik

**Affiliations:** 1 Division of Biopesticides and Environmental Toxicology, SPKCES, Manonmaniam Sundaranar University, Alwarkurichi, Tirunelveli 627412, Tamil Nadu, India; edwin280@gmail.com (E.-S.E.); stanleyrajamsu@gmail.com (V.S.-R.); sivanesh2020@gmail.com (H.S.); heyram.8238@gmail.com (R.R.); 2 Department of Microbiology Division of Virology and Molecular Biology, St. Peters Medical College Hospital and Research Institute, Hosur 635109, Tamil Nadu, India; 3 Department of Biotechnology, St. Peter’s Institute of Higher Education and Research, Avadi, Chennai 600054, Tamil Nadu, India; vasanth.bmg@gmail.com; 4 Department of Botany, V.O. Chidambaram College, Thoothukudi 628008, Tamil Nadu, India; nmcpandian@gmail.com; 5 Department of Biochemistry, School of Life Sciences, St. Peter’s Institute of Higher Education and Research, Avadi, Chennai 600054, Tamil Nadu, India; nrkishnan@gmail.com; 6 Department of Botany and Microbiology, College of Science, King Saud University, Riyadh 11451, Saudi Arabia; huqailasma@gmail.com (A.A.A.-H.); khnfaheema@gmail.com (F.K.); 7 Department of Entomology and Plant Pathology, Faculty of Agriculture, Chiang Mai University, Chiang Mai 50200, Thailand; 8 Innovative Agriculture Research Center, Faculty of Agriculture, Chiang Mai University, Chiang Mai 50200, Thailand; 9 Department of Plant Protection, Faculty of Agriculture Saba Basha, Alexandria University, Alexandria 21531, Egypt; Ahmedabdelfattah@alexu.edu.eg; 10 Department of Zoology, Faculty of Science, Derna University, Derna 417230, Libya; moly2gm@gmail.com; 11 National Institute of Crop Science, RDA, Planning & Coordination Division, 181, Hyeoksin-ro, Iseo-myeon, Wanju-Gun 55365, Jeollabuk-do, Korea; paikch@korea.kr

**Keywords:** andrographolide, RGR, RCR, ECI, ECD, molecular docking

## Abstract

*Spodoptera litura* Fab. is a polyphagous pest causing damage to many agriculture crops leading to yield loss. Recurrent usage of synthetic pesticides to control this pest has resulted in resistance development. Plant-derived diterpenoid compound andrographolide was isolated from the leaves of *Andrographis paniculata.* It was analysed by gas chromatography-mass spectroscopy and quantified by HPLC. Nutritional indices and digestive enzymatic profile were evaluated. Third, fourth and fifth instar larvae were treated with different concentrations of andrographolide. At 3, 6 and 9 ppm-treated concentrations the larvae showed decreased RGR, RCR, ECI, ECD values with adverse increase in AD. The digestive enzymes were significantly inhibited when compared with control. Conspicuously, andrographolide showed pronounced mortality of *S. litura* by inhibition of enzyme secretion and intake of food. The binding ability of andrographolide with CYTP450 showed high affinity with low binding energy. Andrographolide has the potential to be exploited as a biocontrol agent against *S. litura* as an eco-friendly pesticide.

## 1. Introduction

The success of agriculture mainly depends on the yield which is affected by various insect pests causing crop damage. *Spodoptera litura* is a polyphagous pest attacking more than 150 host species, feeding on cotton, tobacco, groundnut, soybean, etc. [1]. It is one of the most economically important insect pests in many countries including India, Japan, China and Southeast Asia [2,3].

Chemical pesticides play a vital role in crop protection. The synthetic chemical pesticides bring many negative effects such as development of resistance in insects, contamination of water bodies, leaving toxic residues etc., Because of this, researchers are looking for alternative method to control pests in an eco-friendly manner. Biologically active substances from plants have the ability to protect from herbivores including lepidopteran pests [4]. They degrade rapidly in the air and moisture and are readily broken down by detoxifying enzymes, thus reducing the risk to non-target organisms [5]. The main advantages of bio-pesticides are they are target-specific, easily biodegradable and safe to living organisms and environment [6,7].

Andrographolide, a natural plant compound from *Andrographis paniculata*, is considered to be a good candidate to control pests since it has many medicinal properties [8,9]. The extract of this herb showed wide range of biological activities [10]. Antimalarial activity against *Plasmodium berghei* [11], and antifeedant activities against *Plutella xylostella* [12], and *Helicoverpa armigera* [13] have been reported.

Food consumption and enzyme activity are mainly affected by secondary metabolites which are deleterious to insects [14,15]; they also bring almost stunted growth of late instars [16]. Damage of peritrophic membrane in the insect gut causes a significant reduction in food utilization [17].

Certain compounds extracted from plants have the ability to affect the enzymatic profile of insect pest. For instance, among them, the proteinaceous inhibitors have the ability to inhibit proteolytic activity and lead to disturbed growth and development [18,19].

Computational docking analysis is mainly used to find the binding ability of the compound to the receptor. The three-dimensional structure of the protein-ligand could serve as a new way to predict the biological functions [20]. The main aim of this study was to find the toxic effects of andrographolide on *S. litura* and its binding affinity with detoxifying enzyme CYP450.

## 2. Results

### 2.1. Analysis of Purified Plant Compound Andrographolide

**Figure 1:**
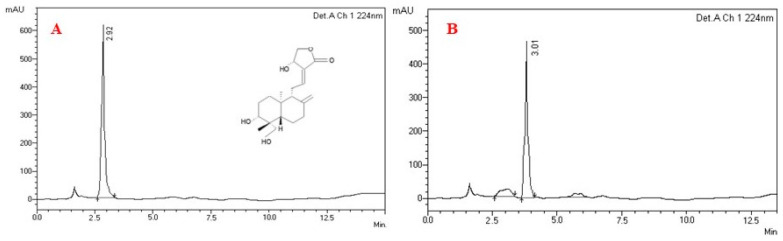
High-performance liquid chromatography (HPLC) chromatogram of standard andrographolide (**A**) along with their chemical structure; (**B**) isolated compound andrographolide.

Our earlier research showed that the GC-MS characterization of crude extracts of *A. paniculata* identified five compounds, with the active fraction being pentadecanoic acid, 13-methyl, phytol, retinoic acid, andrographolide and ergosterol. Among them, Andrographolide was the chief component identified [9]. The isolated plant compound from column chromatography was analysed by HPLC; it had a purity of 88% when compared with standard andrographolide displayed along with chemical structure (Figure 1A,B). The analyzed compound was eluted at the Rt 3.01, which was detected at 224 nm and was compared with standard chromatogram. The andrographolide standard was eluted at the retention time (Rt) of 2.92. This confirmed the isolated compound to be andrographolide.

### 2.2. Food Utilization of S. Litura

The larvae which consumed the food treated with andrographolide showed considerable changes. They showed reduction in the nutritional indices of RCR and RGR at different larval stages of *S. litura*. Reduction in food utilization was dependent on treated concentration. As the food consumption was reduced the larval growth was retarded significantly. A significant reduction (*p* ≤ 0.001) in all nutritional indices was observed at all treatment concentrations. Ingestion and digestion of food were reduced in all instars.

**Figure 2:**
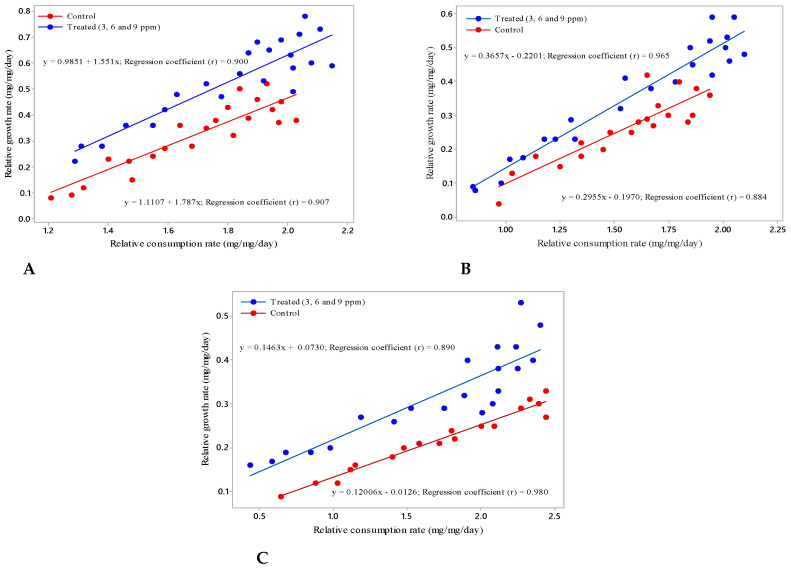
Regression equation and correlation between relative growth rate and relative consumption rate of (**A**) third (**B**) fourth (**C**) fifth instar larvae of *S. litura* fed on leaves containing andrographolide with different concentration.

In the third instar larvae, the RGR (0.28 ± 0.0018 mg/day), RCR (1.63 ± 0.0178 mg/day), ECI (17.19%) and ECD (32.22%) values were influenced by andrographolide (Figure 2A). The approximate digestibility increased (53.37%) when compared with control (48.34%). This extended the larval period with lower RGR and the food retained in the gut region increased the digestibility. The regression coefficient of RCR-RGR for control was (R^2^ = 0.900), and for the treated larvae it was (R^2^ = 0.907).

The fourth instar larvae fed with andrographolide had shown reduced growth and consumption rate. The AD value got increased significantly in treatments and maximize in 9 ppm treatment (63.09%). The ECI (19.08%) and ECD (30.25%) were also significantly reduced in 9 ppm treatment as compared to other treatment and control (Figure 2B). The regression of RCR-RGR for control (R^2^ = 0.965), and the treated (R^2^ = 0.884) were significantly different in the treated diet when compared with control. The larvae were incapable to carry on normal physiological processes as the RCR was very low.

There was a significant reduction in nutritional indices for fifth instar larvae when treated with different concentrations of andrographolide, and the rate declined by the maximum at 9 ppm treatment in ECI (27.75%), ECD (41.29%) and AD (67.33%), respectively. The regression coefficient of RCR-RGR for control was R^2^ = 0.890 and for the treated it was R^2^ = 0.980. This result was significantly different when compared to the control (Figure 2C).

### 2.3. Amylase Activity

**Figure 3:**
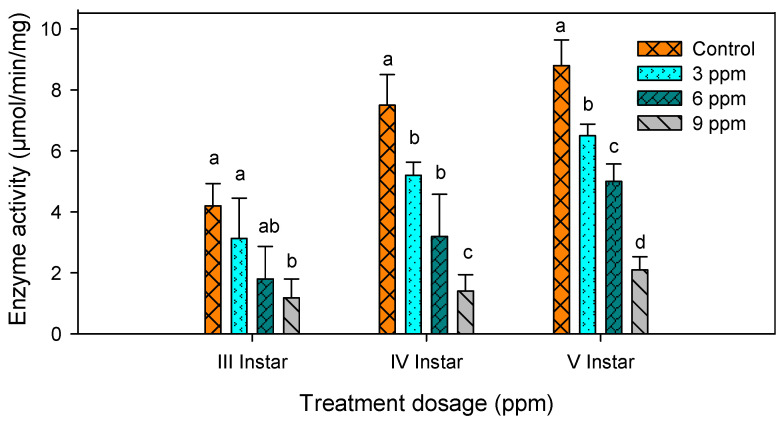
Digestive enzyme amylase activity in third, fourth and fifth instar larvae of *S. litura* after treatment with andrographolide. The data were fitted on a polynomial (regression) model. Means (± standard error (SEM)) followed by the same letters above bars indicate no significant difference (*p* < 0.05) in a Tukey’s test.

Activity of amylase was significantly decreased with increase in concentration when the larvae were fed with different concentrations of andrographolide (Table 1). The present data showed that the compound had suppressed the activity of amylase (Figure 3).

There was a significant reduction in amylase activty in third (*F*_3,16_ = 9.26; *p* ≤ 0.001), the fourth (*F*_3,16_ = 37.04; *p* ≤ 0.001) and the fifth instar larvae (*F*_3,16_ = 36.76; *p* ≤ 0.001).

### 2.4. Lipase Activity

**Figure 4:**
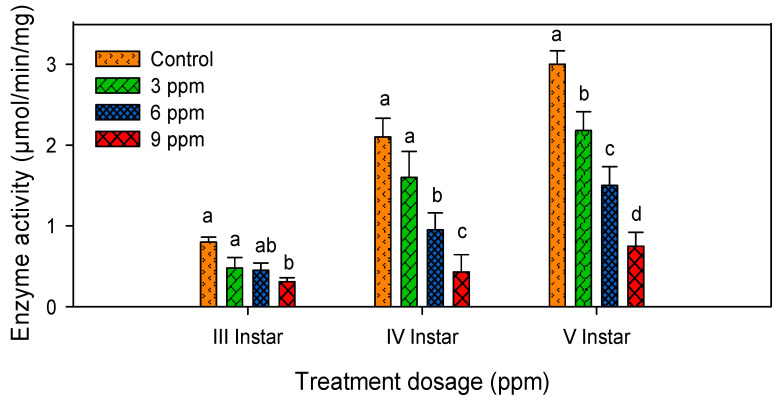
Digestive enzyme lipase activity in third, fourth and fifth instar larvae of *S. litura* after treatment with andrographolide. The data were fitted on a polynomial (regression) model. Means (± standard error (SEM)) followed by the same letters above bars indicate no significant difference (*p* < 0.05) in a Tukey’s test.

Lowest lipase activity was noted in treated larvae when compared with control. Lipase activity was reduced at a maximum (79.5 %) in andrographolide treated larvae (Table 2). It showed significant reduction (*F*_3,16_ = 24.57; *p* ≤ 0.001) in the third instar, (*F*_5,24_ =7.28; *p* ≤ 0.003) in the fourth instar and (*F*_5,24_ = 5.66; *p* ≤ 0.008) in the fifth instar larvae (Figure 4).

### 2.5. Protease Activity

**Figure 5:**
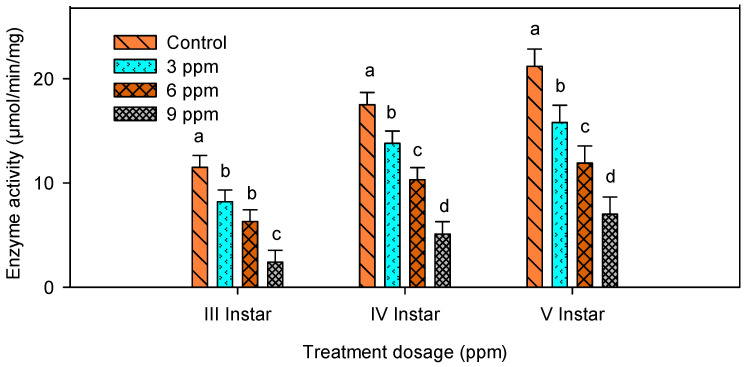
Digestive enzyme protease activity in third, fourth and fifth instar larvae of *S. litura* after treatment with andrographolide. The data’s were fitted on polynomial (regression) model, Means (± standard error (SEM)) followed by the same letters above bars indicate no significant difference (*p* < 0.05) in a Tukey’s test.

Andrographolide-treated larvae showed significant reduction in digestive enzyme activity. Protease activity was low when the larvae were treated with different concentrations of andrographolide (Table 3). Maximum reduction of 79.1% was observed in treated larvae when compared with control (Figure 5). This significant reduction in protease activity reduced the digestibility of food.

The protease activity showed significant differences (*F*_3,16_ = 19.66; *p* ≤ 0.0001) in the third instar, (*F*_3,16_ = 42.72; *p* ≤ 0.0001) in the fourth instar and (*F*_3,16_ = 56.86; *p* ≤ 0.0001), in the fifth instar larvae.

### 2.6. Docking Studies

**Figure 6:**
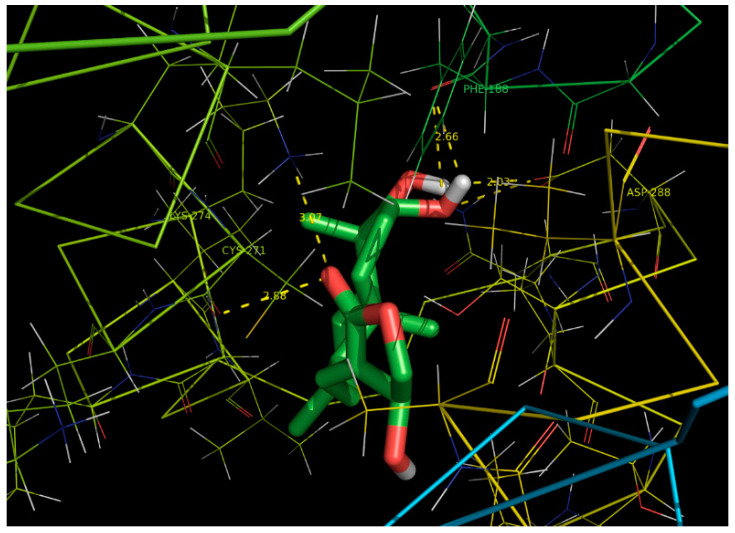
The ligand protein interaction of andrographolide with cytochrome P450 representing the binding pockets for the corresponding amino acid residues.

The docking studies to find the binding affinity of andrographolide to cytochrome P450 of *S. litura* showed very high binding potential. Andrographolide had strong binding interaction with cytochrome P450 (Figure 6). It showed low binding energy suggesting a stable complex formed between the ligand and target protein. The binding regions and binding energy are presented in Table 4.

## 3. Discussion

The use of chemical pesticides for crop protection has increased in developed and growing countries; this has led to various environmental pollution disputes [21]. Synthetic pesticides and their by-products had shown several side effects such as acute and chronic toxicity in natural beneficial insects [22]. These environmental issues urge us to search for ecofriendly pesticides [23].

The plant *A. paniculata* is well known for its biological activity due to the presence of andrographolide. The crude extract of *A. paniculata* had shown antifeedant and antioviposition activity against *P. xylostella* [24]. Hexane and chloroform extract of *A. paniculata* showed 100% mortality at 1000 ppm in *A. subpictus* [25].

RGR and RCR were reduced in andrographolide-ingested larvae when compared with control. Such a reduction was also noticed in *Pierisrapae* (Linnaeus) due to crude leaf extract of *Artemisia annua* and *Achilleamille folium* [26]. The decrease in consumption rate may be due to antifeedant effect of the compound. The same trend was observed in *S. litura* treated with methanolic flower extract of *Chrysanthemum fuscatum* [27] and secondary metabolite treatment of *S. litura* [28,29]. Similarly, Koul et al. [30] observed that aglaroxin A inhibited the food intake. It triggered reduced RGR and RCR with a significant change in the ECI values on both *H. armigera* and *S. litura*. This indicated the toxic substance ingested had exhibited some chronic toxicity. These results were also noticed in *S. litura* when treated with *Trichilia americana* [14]. Andrographolide had shown reduced growth rate, consumption rate, consumption index and conversion of ingested food, but showed higher approximate digestibility. Similar trends were noted in *Glyphodes pyloalis* treated with azadirachtin displayed in the previous research of Khosravi and Sendi, [31], *S. litura* treated with nucleo polyhedro virus, and azadirachtin [32] and 3-*O*-acetylsalannol, salannol and salannin from *A. indica* [33]. Andrographolide acted as a good antifeedant against the lepidopteran pests. Recent studies by Nakhaie et al. [34] also showed the same effect on *S. littoralis* using methanolic extracts of *A. millefolium* and *Teucrium polium*.

Growth efficiency of *S. litura* treated with different concentrations showed significant reduction when compared with control. This growth reduction is due to toxic effect of andrographolide. The same result was noticed by Senthil-Nathan et al. [35] on *Cnaphalocrocis medinalis* and *S. litura* fed with azadirachtin; this reduction was due to the toxic effect of pure limonoid azadirachtin and not by starvation. Decreased larval growth was coupled with lower RGR, because the food was retained in the gut of the larvae for maximum time period with digestibility increment [36].

There was a reduction in digestibility due to ingestion of andrographolide. The reduction in digestibility was noticed by Rath et al. [37] on fifth instar larvae of *Antheraea mylitta* when treated with *Nosema* sp.

The digestive enzymes were reduced when compared with control larvae. According to Broadway and Duffey [38] feeding is essential for the stimulation of enzyme activities. Amylase activity was reduced in andrographolide-treated larvae when compared with control. This result is consistent with the findings of Shekari et al. [39] who found decreased α-amylases activity when *Xanthogaleruca luteola* was treated with *Artemisia annua.* Lipase activity showed considerable reduction in the larvae fed on the diet containing andrographolide. Senthil-Nathan [40] reported reduction in lipase activity in *Cnaphalocrocis medinalis* when treated with botanical insecticide.

The protease activity was significantly lower when compared with control larvae. A similar observation was noticed in many lepidopteran larvae [41]. The compound andrographolide has inhibited the breakdown of peptide bonds in dietary proteins. A similar result was obtained by De-Leo et al. [42] and Macedo et al. [43] when lepidopteran larvae were treated with kunitz. The enzymatic changes in amylase, lipase and protease observed in andrographolide treated larvae were also observed by Senthil-Nathan, [40] on *Cnaphalocrocis medinalis* treated with extracts of *Vitex negundo* and *Azadirachta indica.* The enzymatic reduction by plant-based compounds could easily affect the epithelial cells.

Cytochrome P450 monooxygenases (P450s) of insects are well known for the metabolism or detoxification of plant allelochemicals and insecticides [44,45]. Our present results suggests that the cytochrome P450 enzyme has high binding affinity towards andrographolide. Similar to our finding, Feyereisen [46] found terpenoids had ability to affect the expression of P450 in insects. In vitro study made by Bullangpoti et al. [47] proved that leaf extracts of *M. azedarach* inhibited esterases and P450 enzyme activities.

As the compound had strong binding with different amino acid residues, the activity of the insect was arrested and further functions carried out by the P450 become blocked. Previously Li et al. [48], and Wang et al. [49] demonstrated that P450 was also involved in insect development, reproduction and ecdysteroid degradation.

As an endnote, the dynamic phyto-chemical andrographolide delivers significant shifts in the metabolic enzyme regulation especially against CYP450 and display a crucial role in managing lepidopteran insects.

## 4. Materials and Methods

### 4.1. Isolation of Plant Compound Andrographolide

The leaves of *A. paniculata* were collected at Tirunelveli in the early morning. They were taxonomically identified by Dr. M. Chellappandian, Assistant Professor, PG and Research Department of Botany, V.O. Chidambaram College, Thoothukudi, Tamil Nadu, India. The voucher specimen VOCCBOT-003 was deposited in the herbarium of the college. The leaves were shade dried and powdered. About 500 g of powder was soaked in ethanol to obtain crude extract.

The ethanol crude extract was fractionated using column chromatography and eluted with different gradients of chloroform and methanol. Eluted of 90:10, 80:20, 70:30, 60:40, 50:50 and 40:60. The fraction obtained in 60:40 showed larvicidal activity. It was analysed by GC-MS. This revealed the presence of andrographolide as a major compound [9]. It was further purified by column chromatography and analyzed by HPLC Agilent Technologies LC 8A with C_18_ column (250 mm × 4.6 mm, California, USA). It was also compared with a standard chromatogram of andrographolide.

### 4.2. Insect Rearing of *S. Litura*

*Spodoptera litura* larvae were collected from castor (*Ricinus communis* L Euphorbiaceae) plant in Nagercoil, Kanyakumari district, Tamilnadu, India. Insects were cultured and maintained according to Senthil-Nathan et al. [50]. Larvae were reared in the laboratory on castor leaves. The castor plants were grown in the field and were 1.5–2 months old. For the leaf tests and mass culture, we used mature leaves (75–125 cm^2^) that were removed from the upper third of the plant. Pre-pupae were separated and provided with vermiculture clay as pupation sites. Emerging adult moths were transferred to cages and fed on a 10% sucrose solution fortified with a vitamin mixture to enhance oviposition. Moths were transferred at a ratio of 1 male:2 females to oviposition cages containing castor leaves and covered with muslin cloth for egg laying. The muslin cloths containing eggs were removed daily and eggs present were surface sterilized (to prevent microbial infection) in situ by dipping in 10% formaldehyde solution for 2–5 min and then washed with distilled water. The muslin cloths containing eggs were moistened and kept in plastic containers to allow hatching. All the experiments and cultures were carried out at 28 ± 2 °C, 65% relative humidity, with a 14:10 light: dark cycle.

### 4.3. Food Utilization, Consumption and Nutritional Indices

Food utilization, consumption and nutritional indices of *S. litura* were calculated by the procedure of Senthil-Nathan et al. [50]. Third, fourth and fifth instar larvae of *S. litura* were starved for about 3 h. About 10 larvae were introduced in a container (4 × 4 cm); before that, the initial weights were noted. The castor leaves treated with different concentrations of andrographolide (3, 6 and 9 ppm) and control with 0.1% methanol were air dried. After 24 h, uneaten leaves were weighed and new leaves were introduced. At the end of the day the leaves were weighed and the larvae were dried in oven at 60 °C for 48 h. They were again reweighed to find out percentage of dry weight. Similarly, dry weights of diet and fecal matter were recorded under experimental conditions. The food ingestion was estimated by subtracting the leaves remaining at the end of the experiment from the total dry weight of the leaves. All weights were measured using an electronic balance (Sartorius, Germany).

Nutritional indices of *S. litura* such as:

- Relative consumption rate (RCR) = dry weight of food eaten/duration of feeding (days) × mean dry weight of the larva during the feeding period;
- Relative growth rate (RGR) = dry weight gain of the larva during the period/duration of feeding (days) × mean dry weight of the larva during the feeding period;
- Approximate digestibility (AD) = 100 × (dry weight of food eaten - dry weight of feces produced)/dry weight of food eaten;
- Efficiency of conversion of ingested food (ECI) = 100 × dry weight gain of larva/dry weight of food eaten; Efficiency of conversion of digested food (ECD) = 100 × dry weight gain of larva/dry weight of food eaten.
- Dry weight of feces produced were done according to Waldbauer [51]. Larval growth and food utilization were calculated after 24 h.

### 4.4. Preparation of Enzyme Extract

The treated third, fourth and fifth instar larvae were used to measure enzyme activities. The extraction procedures of Applebaum et al. [52] and Applebaum [53] were followed. Larvae were anaesthetized with ether and the entire digestive tract dissected out in ice-cold insect Ringer’s solution. The Malpighian tubules, adhering tissues, and gut contents were removed. The gut was split into regions, weighed (accuracy in mg) and homogenized in ice-cold citrate-phosphate buffer (pH 6.8) using a tissue grinder for 3 min at 4 °C. The homogenate was suspended in ice-cold buffer and made up to 1 mL. It was centrifuged at 500 rpm for 15 min and the resultant supernatant was used as the enzyme source.

### 4.5. Amylase Activity

Amylase activity was assayed by the dinitro salicyclic acid (DNS) based on the method of Ishaaya and Swirski, [54]. The reaction mixture consisted of 2 mL of 2% freshly prepared starch solution, 1 mL of 0.01 M phosphate buffer (pH 7.2), and 0.25 mL of enzyme extract. After incubating for 60 min at 37 °C, the enzyme activity was terminated by adding 0.4 mL of 3, 5-dinitro salicylic acid reagent. The reaction mixture was maintained at 100 °C for 5 min. Absorbance of the sample was measured at 550 nm against a blank in which the enzyme extract was replaced with deionized water. The amylolytic activity was expressed in terms of the weight of the reducing sugars (glucose) produced by the enzyme action per unit weight of gut, per unit time, using glucose as the standard. The assays were performed five times.

### 4.6. Lipase Activity (EC 3.1.1.3)

The lipase activity was estimated according to Ishaaya and Swirski, [54]. To one ml of gut extract, 0.5 mL of phosphate buffer solution (pH 8.0), and 2 mL of olive oil emulsion were added, shaken well and incubated at 37 °C. The control tube was placed in a boiling water bath for 15 min to destroy the enzyme activity and then cooled. After 24 h, 3 mL of 95% alcohol and two drops of 2% phenolphthalein indicator were added to each tube (control and experimental); the tubes were titrated separately with 0.05 N NaOH solution using a micropipette, and the end point of titration was marked by the appearance of permanent pink color. The experiments were performed five times.

### 4.7. Protease Activity

Protease activity was determined by the Snell and Snell [55] method. We used 1 mL of 50 ppm bovine serum albumin as a substrate. We incubated 1 mL of gut tissue extract and 0.1 mL solution of MgSO_4_ at 37 °C, pH 11.7, for 1 h. The control was made in the same way by adding 1 mL of heat-treated extract. The reaction was terminated by adding 1 ml of 50% trichloroacetic acid. The absorbance was recorded at 600 nm. The experiments were performed five times.

### 4.8. Computational Docking Analysis

The binding ability of andrographolide against cytochrome P450 belongs to the family CYP6B and was determined by Autodock tools (ADT) v1.5.4 [56] and Autodock v4.2 program (Autodock, Autogrid, Autotors).

The chemical structure of cytochrome P450 of *S. litura* was retrieved from the PubChem database http://www.ncbi.nlm.nih.gov/pccompound (accessed on 26 September 2021).

Three-dimensional structure of the selected target protein was recovered from the Protein Data Bank (PDB), (http://www.pdb.org, accessed on 26 September 2021). The Q-site finder was applied to identify the active sites. The selected ligands were docked to target proteins with the molecules treated as a rigid body and the ligands being flexible.

The ligand–protein interactions of the selected compound were analyzed by PyMol molecular viewer (The PyMOL Molecular Graphics System, Version 1.5.0.4 Schrodinger, LLC) and hydrophobic effect of ligands was developed by Pose View [57,58].

### 4.9. Statistical Analysis

Experimental data from mortality were exposed to analysis of variance (ANOVA of arcsine) and data were expressed as a mean of five replicates. Significant differences between treatment groups were examined by Tukey’s multiple range test (significance at *p* < 0.05) using Minitab®17 program. Differences between the treatments were determined by Tukey’s multiple range tests (*p* ≤ 0.05) [59,60].

## Figures and Tables

**Table 1:** Effect of andrographolide on digestive enzyme amylase of *S. litura.*

S. No	Treatments	Third Instar (µmol/min/mg)	Fourth Instar (µmol/min/mg)	Fifth Instar (µmol/min/mg)
1	Control	4.20 ± 0.73 ^a^	7.50 ± 1.03 ^a^	8.80 ± 0.83 ^a^
2	3 ppm	3.00 ± 1.32 ^ab^	5.20 ± 0.29 ^b^	6.50 ± 0.38 ^b^
3	6 ppm	1.80 ± 1.07 ^bc^	3.20 ± 1.38 ^c^	5.00 ± 0.57 ^c^
4	9 ppm	1.18 ± 0.62 ^c^	1.40 ± 0.80 ^d^	2.10 ± 0.94 ^d^

Values followed by different alphabets show significant differences (*p* < 0.05) between control and tratement means.

**Table 2:** Effect of andrographolide on digestive enzyme lipase of *S. litura.*

S. No	Treatments	Third Instar (µmol/min/mg)	Fourth Instar (µmol/min/mg)	Fifth Instar (µmol/min/mg)
1	Control	0.80 ± 0.06 ^a^	2.10 ± 0.94 ^a^	3.00 ± 1.32 ^a^
2	3 ppm	0.48 ± 0.13 ^b^	1.60 ± 0.72 ^ab^	2.18 ± 0.83 ^ab^
3	6 ppm	0.45 ± 0.09 ^bc^	0.95 ± 0.20 ^bc^	1.50 ± 0.87 ^ab^
4	9 ppm	0.31 ± 0.05 ^c^	0.43 ± 0.09 ^c^	0.75 ± 0.17 ^b^

Values followed by different alphabets show significant differences (*p* < 0.05) between control and tratement means.

**Table 3:** Effect of andrographolide on digestive enzyme protease of *S. litura.*

S. No	Treatments	Third Instar (µmol/min/mg)	Fourth Instar (µmol/min/mg)	Fifth Instar (µmol/min/mg)
1	Control	11.50 ± 3.15 ^a^	17.50 ± 2.24 ^a^	21.20 ± 2.77 ^a^
2	3 ppm	8.20 ± 1.09 ^ab^	13.80 ± 0.58 ^b^	15.80 ± 1.03 ^b^
3	6 ppm	6.30 ± 1.44 ^b^	10.30 ± 1.03 ^c^	11.90 ± 1.67 ^c^
4	9 ppm	2.40 ± 1.22 ^c^	5.10 ± 0.56 ^d^	7.00 ± 1.07 ^d^

Values followed by different alphabets show significant differences (*p* < 0.05) between control and tratement means.

**Table 4:** Docked amino acid residues of cytochrome P450 with andrographolide.

Ligand	Protein PDB ID	Binding Amino Acid Residues	Binding Energy (kcal/mol)	Inhibition Constant (uM)	RMSD (Ǻ)	Ligand Efficiency
Andrographolide	CYP6B	PHE`188/O with 42 atoms, CYS`271/O with 10 atoms, LYS`274/NZ with 22 atoms, ASP`288/OD2 with 54 atoms	−6.37	21.42	12.16	0.25

PDB: Protein Data Bank; RMSD: Root Mean Square Deviation.

## Data Availability

The data generated in the current study are available from the corresponding author on request.

## Sample Availability

Samples of the compounds are not available from authors.
